# Progress towards the prevention and treatment of norovirus infections

**DOI:** 10.2217/fmb.13.109

**Published:** 2013-11

**Authors:** Armando Arias, Edward Emmott, Surender Vashist, Ian Goodfellow

**Affiliations:** 1 Division of Virology, Department of Pathology, University of Cambridge, Addenbrooke’s Hospital, Hills Road, Cambridge, CB2 2QQ, UK

**Keywords:** antiviral, calicivirus, gastroenteritis, host factors, norovirus, vaccine, viral polymerase, virus-like particles, VLP

## Abstract

Noroviruses are now recognized as the major cause of acute gastroenteritis in the developed world, yet our ability to prevent and control infection is limited. Recent work has highlighted that, while typically an acute infection in the population, immunocompromised patients often experience long-term infections that may last many years. This cohort of patients and those regularly exposed to infectious material, for example, care workers and others, would benefit greatly from the development of a vaccine or antiviral therapy. While a licensed vaccine or antiviral has yet to be developed, work over the past 10 years in this area has intensified and trials with a vaccine candidate have proven promising. Numerous antiviral targets and small molecule inhibitors that have efficacy in cell culture have now been identified; however, further studies in this area are required in order to make these suitable for clinical use.

## A case for norovirus therapeutic approaches

Noroviruses are now recognized as one of the major causes of gastroenteritis worldwide with an estimated 21 million cases, >70,000 hospitalizations and 800 deaths each year in the USA alone [1]. While low levels of mortality are observed during outbreaks, the overall impact is typically measured in terms of economic losses and disruption to services; for example, foodborne norovirus infections are estimated to result in a loss of US$2 billion per annum [2]. The recent introduction of a vaccine for rotavirus has significantly reduced pediatric gastroenteritis levels in the USA, causing noroviruses to now become the leading cause of pediatric gastroenteritis. While typically an acute disease, norovirus infection often leads to long-term infection in immunocompromised patients and it is now recognized as a significant cause of morbidity and mortality in this population [3,4].

Research in the area of norovirus vaccines and antivirals has increased in intensity in the past 10 years. This is at least partly due to better surveillance mechanisms, improving our understanding of the disease burden, but also due to the availability of assays to study the development of the immune response and the efficacy of small molecule inhibitors. While the efficient culture of human noroviruses (HuNoVs) in immortalized cells has yet to be achieved [5], the development of a norovirus replicon [6], which allows the generation of cell lines stably replicating Norwalk virus RNA, has facilitated many small molecule inhibitors to be tested *in vitro*. The discovery of murine norovirus (MNV) [7], which replicates efficiently in immortalized macrophage cells and has both reverse genetics systems and small animal models available [8], has also enabled the examination of the immune responses to noroviruses as well as the efficacy of inhibitors *in vitro* and *in vivo*.

Noroviruses are members of the *Caliciviridae* family of small positive-sense RNA viruses and in a similar manner to other RNA viruses, they replicate using an error prone mechanism that generates a high degree of genetic diversity. Noroviruses are divided into five genogroups (Gs; GI–GV), with only GI, II and IV causing disease in humans [9]. The error prone replication results in rapid evolution and the generation of new antigenic variants leading to increased diversity and the rapid emergence of new strains capable of evading any herd immunity [9]. Despite these difficulties, substantial efforts have been placed on the generation of control measures, treatments and vaccines, with recent developments in these areas reviewed below.

## Prophylactic & preventative approaches Food testing & treatment

HuNoVs are one of the major causes of foodborne gastroenteritis [1]. Norovirus outbreaks are frequently associated with bivalve shellfish, such as oysters, or with freshly prepared produce such as salads or fruit, generally due to contamination of water with infected fecal matter, or from an infected food handler [10]. Oysters are particularly problematic as they are often eaten raw, and they filter and trap large numbers of particles, serving to concentrate viruses. Outbreaks from contaminated oysters display distinct seasonality and, as such are amenable to testing, detection and forecasting measures [11]. In terms of decontamination methods, high-pressure inactivation (600 MPa) has shown to effectively neutralize infectivity in oysters seeded with HuNoV in volunteer studies, with the added advantage of not involving chemical treatment of the food [12]. Other methods including γ-irradiation, thermal inactivation, steam-ultrasound, UV radiation, chloride or ozone disinfection, and electron beam irradiation have also been tested with varying degrees of success [12]. However, the vast majority of such studies are conducted with norovirus surrogates such as feline calicivirus or MNV, as testing decontamination procedures for HuNoV is difficult owing to the lack of an available cell culture system to detect any remaining infectivity.

### Hygiene & decontamination procedures

Many foodborne norovirus outbreaks can be tracked back to an infected individual. Effective hand washing procedures are essential for the prevention of norovirus spread and for the contamination of foodstuffs; however, the choice of method appears vitally important [13]. Alcohol-based sanitizers are increasingly more popular in healthcare settings; however, they often have limited efficacy against noroviruses with some reports suggesting that their use is in fact a risk factor for norovirus outbreaks in hospitals, although this is currently controversial [1]. Simple mechanical removal of the virus through adherence to proper hand washing techniques appears most effective with the additional incorporation of alcohol-based sanitizers into a hand washing protocol offering minor improvements [14].

Noroviruses remain viable on contaminated surfaces for extended periods of time, and can transfer between surfaces with relative ease [1]. Effective decontamination of norovirus-contaminated surfaces is therefore of vital importance. In the home, the CDC recommends decontamination with diluted solutions containing sodium hypochlorite (bleach). Studies investigating different cleaning regimens for hospitals have shown that detergent-based cleaning is ineffective alone; however, combined detergent and bleach may be effective. For the decontamination of larger areas following outbreaks newer approaches such as use of hydrogen peroxide vapor are under investigation and show promise, although they have only been tested against HuNoV surrogates to date [15].

### Control & prophylactic measures

Control measures are usually implemented for containing and slowing norovirus outbreaks particularly in closed or semiclosed environments such as hospitals, care homes or military bases. Common control procedures during an outbreak include quarantine of infected individuals, enhanced environmental decontamination and enhanced hand hygiene [1,16]. Adherence to control measures are often problematic with only 73% compliance of staff reported during one study [17]. Whilst control measures are clearly important for limiting a norovirus outbreak, further studies are needed to determine their efficacy and apply the appropriate procedures in each occasion.

There are no commercially available prophylactics against norovirus that could be of use during an outbreak. One strategy currently being explored is the use of glycosylated hydrogels [18]. These are crosslinked polymers that can swell to many times their original size upon the addition of water. The incorporation in the hydrogel of human blood group antigens (HBGAs), which are the receptor molecules for HuNoV [19], enables the trapping of viral particles. HuNoV bound to hydrogel would then pass through the gastrointestinal tract and be excreted as normal. A more recently proposed approach involves providing passive immunization by administering antinorovirus antibodies prepared in chicken eggs (IgY) [20].

## Vaccines

The development of effective vaccines has been delayed by the inability to propagate HuNoV in cell culture, preventing the use of viral neutralization assays to monitor the effectiveness of antibody responses [21]. The recent establishment of a receptor-blocking assay whereby the ability of antisera to prevent the interaction of recombinant norovirus virus-like particles (VLPs) with soluble HBGA is examined and has proven to be a suitable alternative to an *in vitro* neutralization assay [22]. Norovirus vaccine development has also been limited by the fact that the immune response to HuNoVs is not well understood [23]. For instance, infection in volunteers with Norwalk virus resulted in a lack of long-term protection against reinfection, suggesting that the immune system is unable to generate a durable response. In addition, given the substantial norovirus strain diversity, it may be difficult to generate a broadly crossreactive vaccine capable of protecting against all norovirus strains. The cost of vaccine development for HuNoV is high, although a recent study has indicated that it would result in substantial cost savings, ≤US$2.1 million over 4 years [24].

Despite the difficulties, there is a strong case indicating the feasibility of a norovirus vaccine [21]. Importantly, the expression of the norovirus major capsid protein VP1 in eukaryotic cells leads to assembly into VLPs, which are antigenically and morphologically similar to native noroviruses. The inoculation of norovirus VLPs *in vivo* results in strong humoral and cellular immune responses [9]. Neutralizing antibodies generated during infection or immunization with VLPs are able to block the binding of viral capsids to their HBGA receptors [22]. Large-scale systems for the preparation of HuNoV VLPs have been developed, including expression in plants such as tobacco and potato, VLPs from which are immunogenic in mice [25].

The route of inoculation is one of the main factors influencing the efficacy of a vaccine. Intranasally inoculated VLPs induced a protective immune response in volunteers subsequently challenged with Norwalk virus, leading to a 47% reduction in the occurrence of gastro enteritis in vaccinated volunteers [26]. High levels of specific IgA antibodies against HuNoV were detected, further supporting this route of inoculation to induce a robust mucosal protection. The advantages of an approach based on intranasal delivery are its ease of administration and the stimulation of mucosal dendritic cells facilitating a local immune response [27]. In fact, a strong mucosal IgA response in gastrointestinal and respiratory tracts has been associated to increased protection against HuNoV [28]. Studies involving an intramuscular route of vaccination in chimpanzees have also provided positive results for norovirus vaccination. Animals inoculated with Norwalk virus VLPs (GI), were protected against subsequent challenge with Norwalk virus, while animals vaccinated with VLPs from a GII norovirus were not [29]. These results highlight the need for the development of efficient bivalent and broadly cross-reactive vaccines.

In summary, the development of vaccines against HuNoV is clearly achievable; however, it is probable that more efficient vaccine formulations are required to overcome the problems associated with the large strain diversity. While promising, vaccines may not be suitable for the treatment of immunocompromised patients in which long-term secretion is common, or for the control of rapidly evolving outbreaks. In both cases, the use of antiviral approaches are likely to be more appropriate.

## Current & future antiviral approaches for the control of noroviruses

### Norovirus replication & life cycle

**Figure 1:**
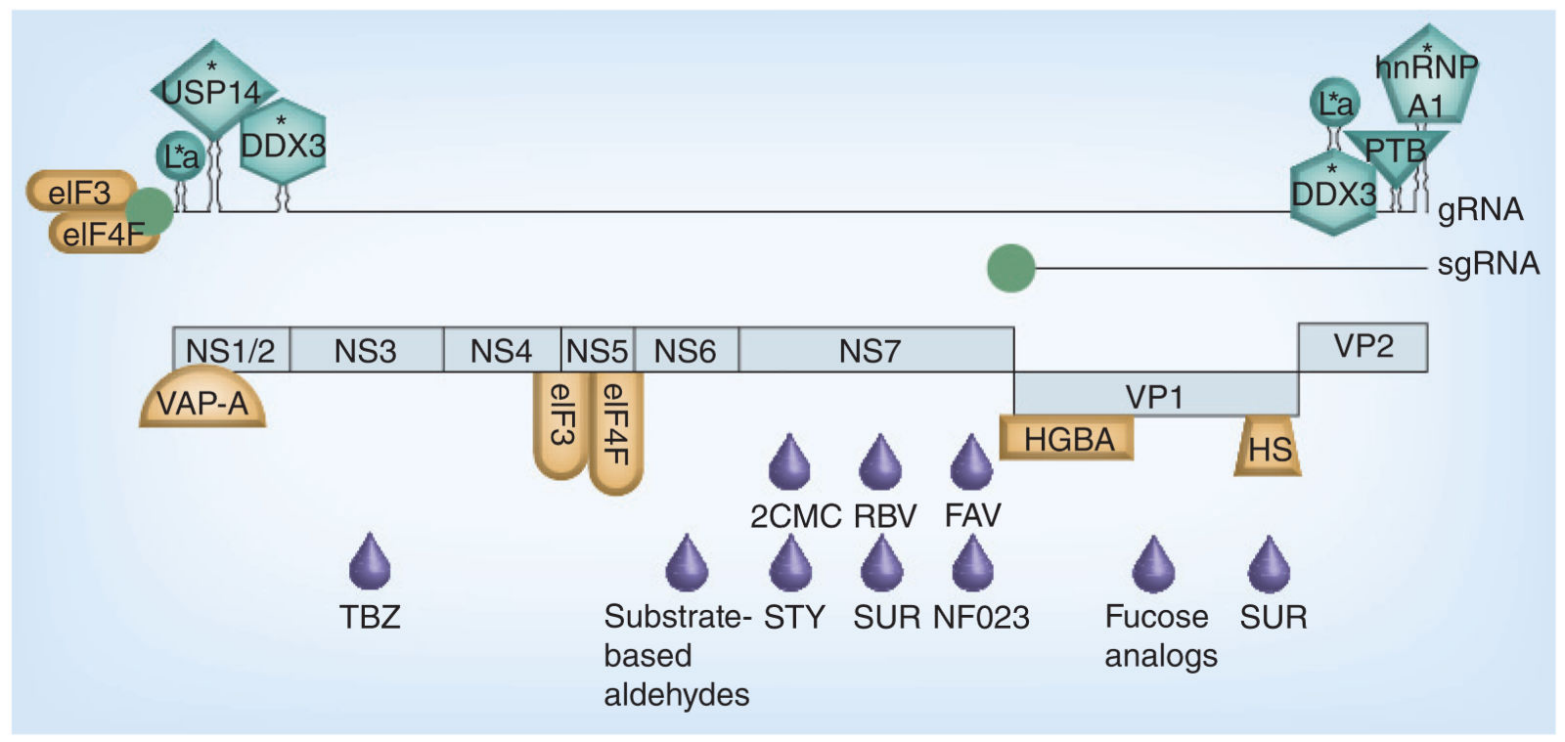
Overview of the known and possible antiviral targets for noroviruses The norovirus gRNA and sgRNA are shown as a larger and a shorter line, respectively, each linked to a VPg molecule (green circle). RNA secondary structures known to form at the extremities of the genome are schematically drawn. The different open reading frames expressed are represented below the genome as light blue boxes. Open reading frame 1 is subdivided into different segments corresponding to the different nonstructural proteins released after proteolysis: NS1/2, NTPase (NS3), NS4, VPg (NS5), protease (NS6pro) and RNA-dependent RNA polymerase (NS7^pol^). Host factors identified to bind to the viral genome extremities are represented as different green shapes. Host factors interacting with viral proteins are represented as different orange shapes. Asterisks highlight host factors for which small molecule inhibitors are available, and their inhibition by these drugs, or downregulation by RNAi, result in decreased norovirus replication. Note that the components shown to interact with the viral RNA genome may interact indirectly via an intermediate protein or proteins. Antiviral compounds targeting different viral proteins are represented below each corresponding region of the genome as purple drop-shaped figures. 2CMC: 2′-C-methylcytidine; FAV: Favipiravir; gRNA: Genomic RNA; RBV: Ribavirin; sgRNA: Subgenomic RNA; STY: Styrylchromones; SUR: Suramin; TBZ: Thiazolobenzimidazoles.

**Figure 2:**
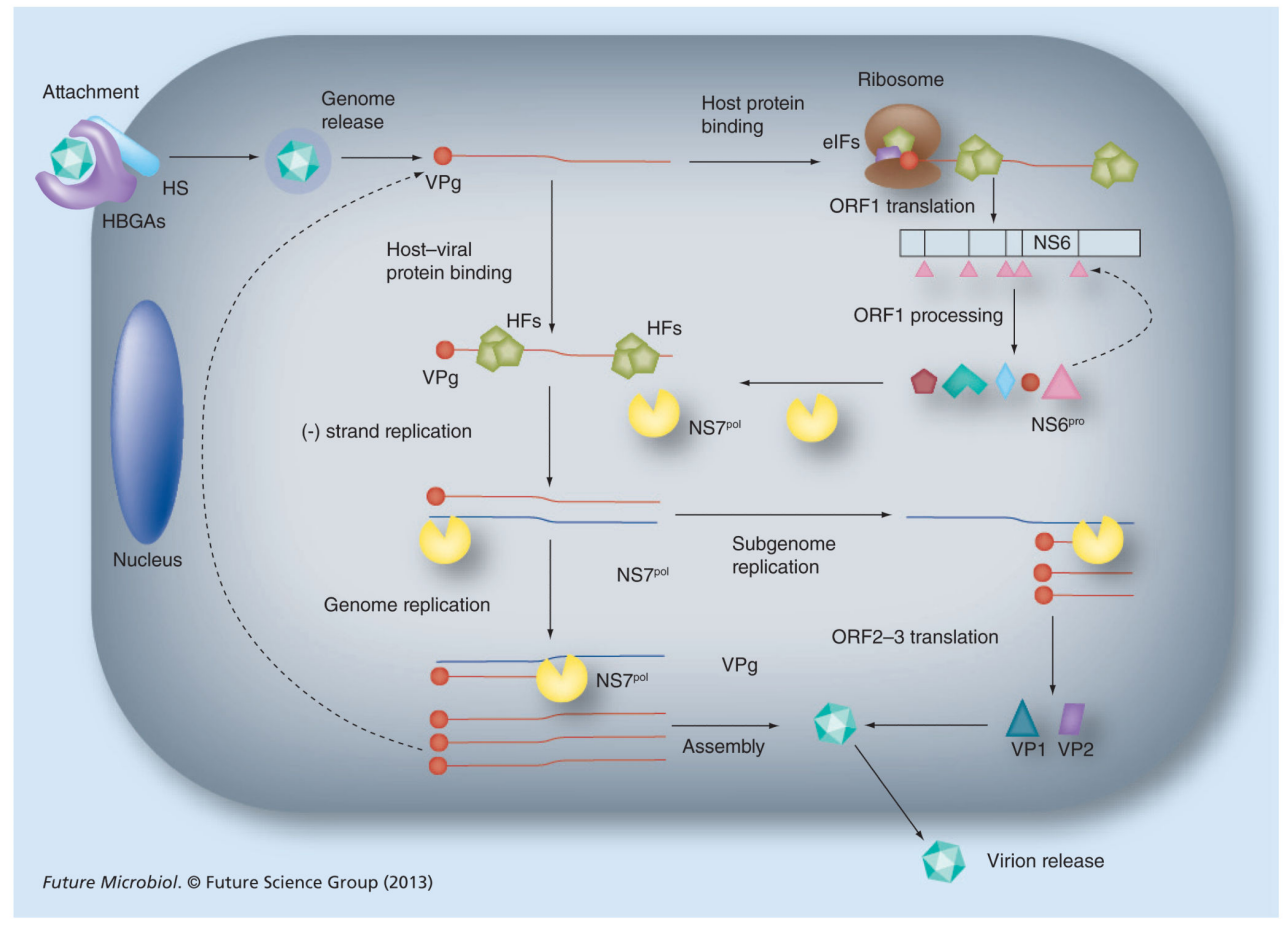
The norovirus life cycle The norovirus infectious particle interacts with HBGA and HS expressed on the cell surface. The attached virion is then internalized and disassembled, leading to the release of the viral genome in the cytoplasm. VPg covalently attached to the 5′ end of the viral genome recruits different HFs, including components of eIF3 and eIF4F, required for cap-independent protein translation, permitting the expression of ORF1. ORF1 is proteolytically cleaved by the viral protease NS6^pro^ (and its precursors). Mature nonstructural proteins released after proteolysis promote the replication of the viral RNA; VPg acts as primer of viral RNA synthesis, remaining covalently attached to the viral genome after replication; the viral RNA-dependent RNA polymerase NS7^pol^ catalyze the synthesis of viral RNA in a process assisted by the helicase-homologous viral NTPase (NS3). The binding of several host factors (HFs) such as La protein, DDX3, hnRNP A1 and PTB to the viral genome is critical for the efficient virus replication. The replication of a subgenomic RNA leads to the generation of shorter viral RNA products that are translated in the major and minor capsid proteins VP1 and VP2 (ORF2 and ORF3, respectively). Newly synthesized genomes are encapsidated in mature particles that are released into the extracellular environment. HBGA: Histo blood group antigen; HF: Host factor; HS: Heparan sulfate; ORF: Open reading frame.

Antiviral strategies against HuNoVs can target many aspects of the virus life cycle; viral proteins or cellular proteins directly, or processes required for virus replication (Figure 1, Tables 1 & 2). The development of such therapies requires an in-depth knowledge of the norovirus life cycle (Figure 2), yet the inability of HuNoV to be efficiently propagated in cell culture has resulted in a limited understanding of this process. A growing body of literature exists on MNV and other members of the *Caliciviridae* family, where parallels can be drawn [8]. Studies on other positive-strand RNA viruses may also provide potential insights into antiviral approaches that may be applicable to HuNoV.

HuNoV interacts with HBGAs and, although binding to HBGA is not sufficient to enter cultured cells, it is thought that this interaction is critical for virus internalization and subsequent infection [9,19]. HBGAs are complex carbo hydrate molecules present in the surface of red blood cells, mucosal epithelia and also in different body fluids (i.e., milk and saliva). HuNoV capsids interact with different families of HBGAs including ABO, secretor and α2,3-sialylated carbohydrates of the type 2 chain (e.g., sialyl-Lewis X). Individuals harboring a single polymorphism in the *FUT2* gene are less susceptible to HuNoV infection, further supporting the hypothesis that HBGAs are the receptor molecules for HuNoV [9,19]. The *FUT2* gene encodes for a α1,2-fucosyl transferase that catalyzes the fucosylation of HBGAs and is responsible for the secretor blood type [19]. Nonsecretor individuals present a polymorphism in the *FUT2* gene resulting in an enzyme unable to fucosylate H-type backbones in HBGAs. Although the interaction with HBGA is required for virus entry, it is thought that other cellular cofactors are required as overexpression of *FUT2* does not rescue infectivity in cells that are competent for HuNoV replication [30]. Recent investigations suggest that HuNoV binding to cells and internalization *in vivo* can also occur independently of HBGA, suggesting the participation of other receptor molecules [31]. In particular, it has been demonstrated that HuNoV capsids interact with heparan sulphate, a cell membrane glycosaminoglycan, which might have a role in cell entry [32]. By contrast, MNV uses the ganglioside GD1a as an attachment ligand for infection of permissive cells, although the relevance of this molecule for HuNoV has yet to be determined [33].

The processes following HuNoV capsid internalization are largely unknown owing to the absence of efficient cell culture systems supporting infection. Studies with the related norovirus MNV demonstrated that virus internalization is dependent on cholesterol and dynamin in a clathrin- and caveolae-independent pathway [34,35].

Once the viral genome (a positive strand RNA molecule 7–8 kb in length) is released in the cytoplasm, viral proteins are synthesized (Figure 2). The HuNoV genome contains three open reading frames (ORFs). ORF1 encodes a polyprotein that, after proteolytic processing, results in the production of six or seven mature nonstructural proteins (NS1–7). These include NS7^pol^, the viral RNA-dependent RNA polymerase (RdRp) responsible for viral RNA synthesis, as well as NS6^pro^, the viral protease that catalyzes the proteolytic processing of ORF1. Translation of structural proteins VP1 (ORF2) and VP2 (ORF3) occurs from a subgenomic RNA produced during virus replication (Figure 2). The process of viral protein translation in noroviruses relies on the recruitment of eukaryotic translation initiation factors to the virus encoded VPg protein (NS5) covalently linked to the 5′ end of the viral RNA. A specific interaction between VPg and eIF3 and/or eIF4E has been demonstrated and may contribute to viral translation initiation [36,37].

Replication of norovirus and other caliciviruses, as previously reported for picornaviruses, occurs in or on intracellular membranes, which are extensively rearranged during virus infection [38,39]. MNV replication complexes can be found associated with double membrane vesicles, originating in the perinuclear region colocalizing with early and late secretory pathway components [39]. Viral replication takes place adjacent to the microtubule organizing center, and utilizes microtubules to position the replication complex, as their chemical disruption reduces viral replication [40]. For HuNoV there is limited information regarding intracellular rearrangements associated to virus replication although it has been suggested that the replication complex is associated to membranes that originated from the disassembly of Golgi [38].

### Strategies targeting viral entry

**Figure 3:**
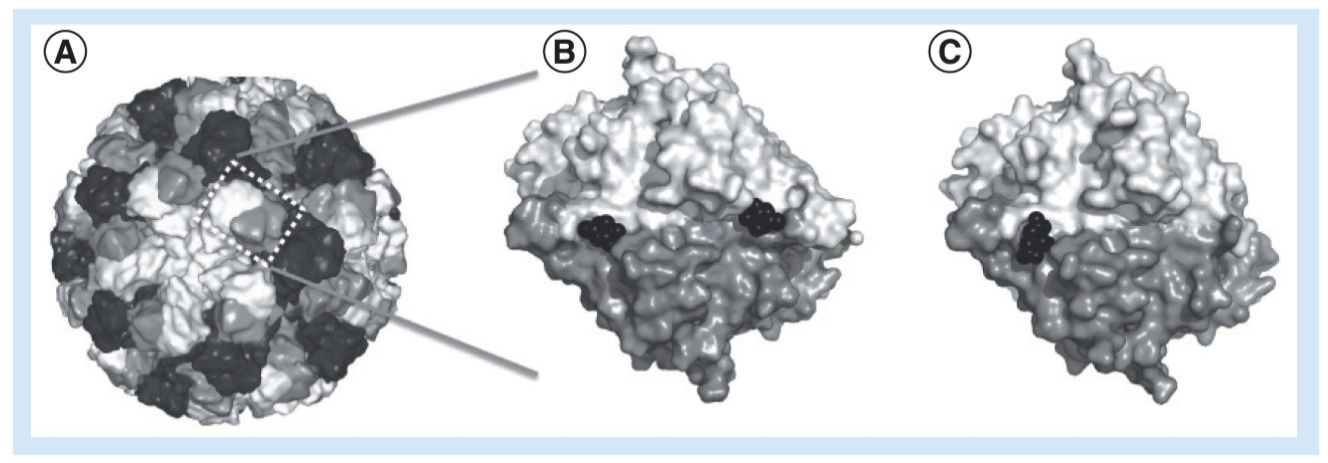
Structure of the viral capsid in complex with natural fucose substrate and citrate inhibitor **(A)** Structure of human norovirus capsid: 60 asymmetric units are assembled to form a mature capsid. Each asymmetric unit contains three copies of VP1 represented in a different grayscale. **(B)** Top view of a human norovirus VP1 dimer bound to two fucose residues (black), the natural binding substrates of the capsid. **(C)** A similar representation as in **(B)**, but illustrating the interaction with citrate, a fucose analog inhibitor, interacting with the fucose-binding pocket. For the modelling of the full norovirus capsid, the program Protein Workshop and the structure available for Norwalk virus (PDB: 1IHM) has been used. PyMol program has been used for the representation of VP1 dimers bound to fucose and citrate (human norovirus Vietnam strain 026, PDBs: 3ONY and 3RY8).

Based on the extensive knowledge on the adhesion of HuNoV capsid to different carbohydrates on the surface of the cell [9,19], multiple approaches targeting viral entry have been proposed. HuNoVs maintain highly conserved residues in the HBGA interaction surface; however, this conservation is only maintained among members of the same genogroup [19]. In GII viruses, the conserved structural region involved in the interaction with α1,2-fucose present in HBGA is located in the outer dimeric capsid interface; while in GI viruses, the interacting surface with HBGA molecules comprise residues from a single capsid monomer [19]. Given the large degree of conservation of capsid interacting surfaces, antiviral approaches have been focused on the design of specific molecules against them (Figure 3).

Examples of carbohydrate analogs with potential antiviral activity are citrate and other glycomimetics since they resemble the molecular structure of fucose (Figure 3) [41]. The identification of novel antiviral compounds targeting the capsid has also been attempted with a novel approach based on library screening, x-ray crystallography and nuclear magnetic resonance [42]. This method has been instrumental in the identification of two new antiviral candidates with high affinity for the fucose binding pocket of Norwalk virus. Standard screening studies involving >5000 synthetic small molecules and enzyme immune assay have also been applied successfully with 14 different compounds identified to effectively inhibit HuNoV capsid binding to at least one of the members of the HBGA ABO family [43]. Since HuNoV capsid binds heparan sulphate, another possible field of investigation would be the use of heparan sulphate analogs such as heparin, suramin and other heparan derivatives [32]. The observation that suramin also targets the viral polymerase (discussed later), further supports its use as a potential therapeutic approach.

One feasible possible approach yet to be followed would be the identification of therapies targeting α1,2-fucosyl transferase encoded by the *FUT2* gene with the aim of transiently reducing HBGA fucosylation. Nonsecretor individuals are naturally less sensitive to infection [9], supporting the hypothesis that transient reduction in the fucosylation may reduce susceptibility to infection.

### Inhibition of viral protein production

#### VPg-host factor interactions implicated in viral translation

Genomic and subgenomic norovirus RNAs recruit host factors for their translation via the viral VPg protein, which is covalently bound to the 5′ end of the viral RNA [36,37]. The use of specific drugs aimed to disrupt these interfaces could provide an attractive antiviral therapy. HuNoV VPg is known to interact with eIF3 and components of the eIF4F complex, thus a possible approach would consist of targeting either of these interactions with small molecules that disrupt their interaction with the norovirus VPg protein but not host cell factors. Hippuristanol is an encouraging example of the existence of drugs that specifically affect virus translation with lower toxicity for the cell. It is a marine compound identified during the screening of a panel of small molecules with affinity for the C-terminus of eIF4A. Hippuristanol inhibits translation of both MNV and feline calicivirus (FCV) and appears to be less toxic for cells than for the virus [44]. Hippuristanol has been recently used to control tumor growth in mice, supporting its use *in vivo* [45]. Other approaches targeting translation initiation include the development of drugs against host factor interacting surfaces in VPg that could now be facilitated by the recent description of MNV and FCV VPg protein structures [46].

#### Viral protease inhibitors

Norovirus ORF1 translation produces a large polypeptide that is cleaved to release the various mature nonstructural proteins in a process carried out by the viral NS6 protease (and its precursors). The recent resolution of the HuNoV NS6^pro^ crystal structure has allowed the identification of new inhibitors based on the specific recognition of the peptide substrate by the protease [47]. A series of products, including bisulfite adducts, dipeptide and tripeptide aldehydes, ketoamides and ketoheterocycles have been synthesized and shown to elicit a strong inhibitory activity against NS6^pro^ *in vitro* and reduced virus replication in cell culture [47,48]. The inhibition of protease activity is an approach widely used for other viral infections [48], which in the case of noroviruses, results in reduced levels of the mature nonstructural proteins required for viral replication.

#### Targeting the viral RNA: RNAi & phosphorodiamidiate morpholino oligomers

Targeting the norovirus RNA genome directly has also been investigated as a method of regulating virus replication. Phosphorodiamidate morpholino oligomers (PMOs) inhibit protein expression of a target molecule by annealing in a Watson–Crick conformation, causing the steric blockade of protein translation. Peptide-conjugated PMOs (PPMOs) directed against the first AUG region in norovirus *ORF1* gene are effective in inhibiting HuNoV and MNV replication in cell culture [49]. PMOs are similar to DNA oligonucleotides, but they are soluble in water and highly resistant to degradation making them suitable for treatments *in vivo*. The use of siRNA molecules to target replication is more efficient than PPMOs; PPMOs and siRNAs targeting the equivalent genomic sequence in FCV showed that siRNA elicited inhibition 50-fold larger than PPMOs [50]. These promising approaches based on siRNA or shRNA molecules may, however, require technical improvements to achieve efficient delivery *in vivo* [51].

#### Interferon

Type I and II interferons elicit a robust anti viral response against HuNoV and MNV, which appears to be due to direct inhibition of virus translation [52,53]. Norovirus replication inhibition is stronger when interferon treatment is combined with ribavirin (RBV), as discussed below [52]. Given that the combination of RBV and interferon is currently used for the clinical treatment of HCV infections, it is likely that the use of the same combination may be effective against noroviruses *in vivo*. However, to date, the therapeutic use of RBV or interferon for the treatment of norovirus infection in humans is yet to be described.

### Inhibition of viral replication

#### Targeting initiation of replication by VPg

Norovirus genome replication requires the protein primer VPg to initiate viral RNA synthesis [54]. Preceding the initiation of viral RNA replication, VPg must be guanylated in a step carried out by the virus polymerase NS7^pol^ or its precursor NS6–NS7 [55]. This guanylated-VPg product is then used as a primer for viral RNA synthesis. Accordingly, it may be possible to design specific nucleoside analogs to compete with GTP to inhibit VPg-nucleotydylylation, or to be incorporated but prevent the subsequent elongation of viral RNA synthesis. For example, in picornaviruses, 5-fluorouracil triphosphate functions as an inhibitor of VPg-primed RNA synthesis [56]. The 5-fluorouracil triphosphate is bound to VPg with higher affinity than uridine 5′-triphosphate, the natural nucleotide typically incorporated, and this binding inhibits the formation of long VPg-primed RNA polymers, suggesting that its antiviral activity is partly due to the blocking of viral RNA synthesis initiation [56].

#### Inhibition of viral RNA synthesis by nucleoside analogs

Typically, the RdRp of RNA viruses are selected as key targets of many antiviral compounds, including nucleoside and non-nucleoside analogs, as they play a central role in the virus life cycle. Several nucleoside compounds have activity against noroviruses in cell culture, opening up their possible application as therapeutic compounds. RBV, a purine analog that has been found to possess antiviral activity against a vast number of different viruses [57], also works against HuNoV and MNV in cell culture [52]. RBV is phosphorylated by cellular enzymes into RBV mono-, di- and tri-phosphate and exerts its broad antiviral effect through various mechanisms: competitive inhibition of inosine monophosphate dehydrogenase, which reduces the intracellular concentrations of guanine nucleotides; inhibition of viral RNA-dependent RNA polymerases; inhibition of mRNA cap formation; enhancement of antiviral immune responses; and lethal mutagenesis of viral quasi species as a result of incorporation of RBV monophosphate into viral RNA (reviewed in [57]). For MNV and the novovirus replicon, RBV antiviral activity is associated with a decrease in GTP levels since the complementation with GTP reduces its antiviral activity. Mycophenolic acid, a non-nucleoside inosine monophosphate dehydrogenase inhibitor, also affects norovirus replication further supporting that norovirus is affected by imbalances in the NTP pools. There is no evidence however, for increased mutation frequencies in noro virus genomes after RBV treatment, suggesting it does not elicit antiviral activity through lethal mutagenesis [52].

A strong inhibitory activity on MNV replication in cell culture was observed using 2′-C-methylcytidine (2CMC) [58], a drug that was initially developed to treat HCV. Recent studies have shown that 2CMC and other derivatives are also efficient in the control of HuNoV replication in cell culture [59]. Concerns over the use of 2CMC have been raised owing to the observation that some toxicity occurred in patients treated for HCV [60]. New derivatives of 2CMC with reduced toxicity in patients are currently under investigation [61], which could also hold promise against norovirus infections.

A recent study with MNV has also supported the use of favipiravir, a nucleoside analog with some homology to RBV, to treat norovirus infections [62]. Favipiravir is similar to RBV in that it is effective against a broad range of viruses and increased mutation frequencies associated have also been reported [63]. Other nucleoside analogs, 2′-arauridine and 3′-deoxyuridine, have also been found to inhibit the viral polymerase activity *in vitro* [50]; however their efficacy in cells or *in vivo* remain to be tested.

#### Non-nucleoside analogs targeting the viral polymerase

Apart from nucleoside analogs, where inhibition is directed to the catalytic site of the viral polymerase, other non-nucleoside compounds have been found to inhibit the norovirus RdRp, in particular, suramin and NF023 [64]. Enzymatic assays have confirmed that suramin and NF023 are inhibitors of HuNoV and MNV RdRps, with IC_50_ values in the nanomolar range. Importantly, suramin is a drug currently used in the clinical treatment of sleeping sickness caused by *Trypanosoma* [65]. In addition to its capacity to inhibit viral RNA replication, suramin has been found to inhibit HuNoV capsid binding to heparan sulphate [32]. The styrylchromones are a new class of flavonoid compounds found to elicit antiviral activities against a broad spectrum of RNA viruses, including MNV [66]. Evidence indicates that they might be targeting the viral RdRp, although the precise mechanism of action is not yet known.

#### Inhibition of viral NTPase activity

The norovirus NTPase (NS3) shares homology with other viral NTPases previously described, such as HCV NS3 and picornavirus 2C. These molecules are classified in the superfamily III of RNA helicases and it is believed that they catalyze the hydrolysis of nucleoside triphosphates to unwind the viral nucleic acids during replication, although no evidence for helicase activity has been reported [67,68]. Nonetheless, the inhibition of viral NTPase activities results in the concomitant inhibition of viral RNA synthesis. Although no inhibitors have been identified against the norovirus NS3, thiazolobenzimidazoles inhibit the replication of several different picornaviruses, by targeting the viral NTPase 2C [69].

#### Targeting viral RNA-interacting host factors involved in virus replication

The untranslated regions in RNA virus genomes normally recruit multiple host factors to the viral RNA genome that play important roles in viral translation and replication. In noroviruses the untranslated regions are extremely short but the coding and noncoding regions in genomic and subgenomic RNA are known to fold into highly-ordered secondary structures that interact with multiple host factors including proteins La, PTB, PCBP, DDX3 and various hnRNPs among others [70,71]. Although the precise role of these proteins in the norovirus life cycle is yet to be determined, they are probably involved in viral RNA replication and genome circularization, a process that is thought to be required for many RNA viruses [72]. So far, no antinorovirus strategies have been investigated based on targeting these proteins, but as small molecule inhibitors exist for some of them, further studies in this area are warranted. For example, the identification of small inhibitory molecules targeting DDX3 has opened the possibility of targeting norovirus infections *in vivo* as specific downregulation of DDX3 protein levels in cells results in the inhibition of MNV [71]. Supporting this possibility, DDX3 inhibitors have shown antiviral activity against HIV in cell culture [73]. DDX3 is a multifunctional host cell RNA helicase implicated in the life cycle of a number of viruses [74]. DDX3 contributes to the innate immune response, but also to host cell translation initiation via interaction with eIF4F and eIF3 [75].

The La protein interacts with HuNoV RNA and reduction of the cellular levels of La also affects norovirus replication in cells [70,71]. Downregulation of cellular La protein levels by small molecule inhibitors has been recently found to elicit antiviral activity against HBV [76], supporting its testing against noroviruses. La is an RNA binding protein originally identified as an autoantigen in diverse autoimmune syndromes and is typically involved in the maturation and translation of some cellular mRNAs [77]. In RNA viruses, La participates in the regulation of inositol-requiring enzyme substrate-driven translation from a variety of different positive-strand RNA viruses; however, its role in norovirus replication has yet to be elucidated.

We have also identified PTB as an important factor associated to norovirus RNA and its binding to a pyrimidine-rich tract in the 3′ terminal stem–loop contributes to virulence in the host [78]. Downregulation of PTB protein levels in cell culture also results in decreased MNV replication [71]. Whilst the function of PTB remains to be fully elucidated, work with FCV, a related member of the *Caliciviridae*, indicated that PTB plays a negative role in viral translation, possibly regulating the shift between translation and replication [79].

hnRNP A1 has also been reported to interact with MNV RNA [71]. The hnRNPs are typically involved in the metabolism of cellular precursor mRNAs [80]. Antitumor drugs targeting hnRNP A1, such as camptothecine and derivatives like 9-nitrocamptothecine [81], could be tested for the treatment of norovirus infections.

### Targeting factors involved in cellular rearrangements associated with infection

#### Inhibition of ubiquitinases & cellular stress response

The cellular deubiquitinase (DUB) USP14 has recently been shown to be involved in HuNoV and MNV replication, and its specific downregulation or inhibition results in reduced virus replication in cell culture and *in vivo* [82]. USP14 interacts with the inositol-requiring enzyme 1, a central protein in the activation of the unfolded protein response (UPR) as a result of cellular stress. USP14 has been also identified to interact with norovirus 5′ genomic RNA extremity, although whether this is a direct RNA–protein interaction or one mediated via an intermediate interacting protein remains to be determined [71]. A small molecule inhibitor of several DUBs including USP14 has been found to inhibit both MNV and novovirus replication, and this inhibition is associated to the activation of UPR in an inositol-requiring enzyme 1-dependent mechanism. The authors have proposed that the inhibition of cellular DUBs leading to the subsequent UPR constitute a novel target approach to block viral infections [82].

#### Membrane rearrangement & trafficking host factors

HuNoV NS1–2 protein, p48, interacts with VAP-A, a SNARE-binding protein with an important role in cellular vesicle transport regulation. It is believed that this interaction is responsible for localizing NS1–2 to intra cellular vesicles in cells and preventing normal intracellular protein trafficking [38]. Expression of HuNoV NS1–2 is sufficient to block the expression of membrane proteins at the cell surface. Therapies based on disrupting the interaction between these proteins or regulating the membrane rearrangements have yet to be explored but may prove a tractable approach worth considering. For example, botulinum toxin type B is a drug that targets VAMP-1 and has been approved for the treatment of cervical dystonia [83]; however, the issue of toxicity and the ability to deliver to the site of virus replication remains a significant obstacle to be overcome.

## Future perspective

The socio-economic impact of norovirus infection is now well established, thus the case for the development of effective vaccines and antiviral approaches is strong. Whilst there are many inhibitors that have proven efficacy against noroviruses in cell culture and at least one that appears to work in the mouse model for MNV, none have made it through to clinical use for the treatment or prevention of norovirus infection. As with all therapies targeting intestinal pathogens, there is a significant problem associated with the delivery of the inhibitor to the site of replication, as well as stability in this rather harsh environment. Subsequently, there are a number of significant hurdles that must be overcome for the use of these inhibitors in a clinical setting. An area that has yet to be fully explored is the use of inhibitors that are currently in clinical use for other RNA viruses. RBV is widely used for the treatment of viral infections and has been shown to be effective in cell culture against HuNoV, yet clinical use in the treatment of chronic norovirus infections has yet to be described. Given the conserved nature of RNA synthesis, the viral RNA polymerase provides a particularly attractive target as numerous nucleoside and non-nucleoside inhibitors are under trial for other RNA viruses such as HCV. Clearly further studies in this area are required; these inhibitors hold great promise for the treatment of numerous RNA virus infections, including noroviruses.

As discussed above, a major limitation to the development of effective control measures has been the lack of a fully permissive cell culture system and small animal model for HuNoVs. The momentum generated by researchers in the field has resulted in numerous developments in these areas, yet a licensed vaccine or clinically approved antiviral remains elusive. The development of an *in vitro* culture system and an animal model that recapitulates all aspects of the disease observed in humans would prove invaluable and should remain a high priority. A better understanding of the correlates of protection would also enable better vaccine design and could lead to the generation of a broadly protective vaccine that may at least produce short-term immunity in particularly vulnerable or regularly exposed individuals. Until such a time, the use of effective quarantine and hygiene measures remain the only alternative for the control of norovirus outbreaks and provide a way of minimizing the impact of these important pathogens.

## Figures and Tables

**Table 1:** Compounds targeting host factors required for norovirus replication.

Compound	Cellular target	Inhibitory activity
FE15 and derivatives	DDX3	Inhibits ATPase activity of DDX3. Not tested against noroviruses, but antiviral activity proven for HIV
Hippuristanol	eIF4A	Inhibits activity of eIF4A and potently inhibits MNV in cell culture
WP1130	USP14	Inhibits virus replication *in vivo*
HBSC compounds	La protein	Not tested against noroviruses, but antiviral activity shown for HBV
Camptothecine and derivatives	hnRNP A1	Not tested against noroviruses

MNV: Murine norovirus.

**Table 2:** Antiviral molecules directed against viral proteins or RNA.

Compound	Viral target	Inhibitory activity
2′-C-methylcytidine and derivatives	NS7 polymerase	Replication in cell culture inhibited
Fapivirapir	NS7 polymerase	Replication in cell culture inhibited
Ribavirin	NS7 polymerase	Inhibits replication in cell culture through imbalances in NTP pools
Mycophenolic acid	NS7 polymerase	Inhibits replication in cell culture through imbalances in NTP pools
Styrylchromones	Possibly NS7	Replication in cell culture inhibited
2′-arauridine and 3′-deoxyuridine	NS7 polymerase	Polymerase inhibition *in vitro*
NF023	NS7 polymerase	Polymerase inhibition *in vitro*
Suramin	NS7 polymerase	Polymerase inhibition *in vitro*
Suramin	Capsid (VP1)	Reduced binding to intestine cells
Heparin	Capsid (VP1)	Reduced binding to intestine cells
Citrate and other fucose analogs	Capsid (VP1)	Inhibits binding to HBGAs *in vitro*
Substrate-based aldehydes	Protease (NS6)	Inactivate protease by covalent binding *in vitro*
PPMOs	Viral RNA	Inhibit translation by antisense annealing in cell culture
siRNAs	Viral RNA	Activate cellular RNAi response against viral genome
Interferon	Viral translation	Replication in cell culture inhibited

HBGA: Histo blood group antigen; PPMO: Peptide-conjugated phosphorodiamidate morpholino oligomer.

## Executive summary

### A case for norovirus therapeutic approaches

■ Noroviruses are a major cause of gastroenteritis.
■ Infection results in >70,000 hospitalizations and 800 deaths in the USA per annum.
■ Economic losses exceed US$2 billion per annum in the USA alone.
■ Noroviruses are a very diverse group of viruses that replicate using an error prone mechanism, facilitating the regular emergence of new antigenic variants.
■ Chronic long-term shedding of virus is common in the immunocompromised.

### Prophylactic & preventative approaches

■ Noroviruses are stable in the environment and difficult to remove.
■ Current control measures involve quarantine and increased hygiene practices.
■ Alcohol hand sanitizers are generally poorly effective against noroviruses.

### Vaccines

■ Expression of the major capsid protein in eukaryotic cells produced virus-like particles that are antigenically identical to infectious norovirus.
■ Immunization with virus-like particles produces a protective immune response, which is genogroup specific.
■ The development of broadly crossprotective immune responses has yet to be achieved.

### Current & future antiviral approaches for the control of noroviruses

■ The norovirus life cycle is poorly understood owing to an inability to culture human norovirus in immortalized cells.
■ Recent work has developed human norovirus replicons and identified murine norovirus as the only norovirus that can replicate efficiently in cell culture.
■ Numerous antivirals targeting many aspects of the virus life cycle have now been identified but very few have been tested in animals.
■ Targeting the viral RNA polymerase or protease holds promise given the effectiveness in other viral systems.
